# Synergistic Co-Delivery of siFGF2 and Doxorubicin via QTPlus Nanoparticles for Enhanced Breast Cancer Therapy

**DOI:** 10.3390/pharmaceutics18050589

**Published:** 2026-05-10

**Authors:** Xiaohan Xia, Zhongkun Zhang, Jingjing Zhang, Kaixin Feng, Yufei Wang, Robert J. Lee, Siyu Yao, Min Wu

**Affiliations:** 1 Division of Chemical Engineering, School of Chemistry and Chemical Engineering, Southeast University, Nanjing 211189, China; 2 Department of Pharmacology, School of Medicine, Southeast University, Nanjing 210009, China; 3 Department of Medical Oncology, Dana-Farber Cancer Institute, Harvard Medical School, Boston, MA 02115, USA; 4 The Whiteoak Group, Inc., Rockville, MD 20855, USA; robert.lee@thewogroup.com; 5 Key Laboratory of Environmental Medicine Engineering, Ministry of Education, School of Public Health, Southeast University, Nanjing 210009, China; siyuyao@seu.edu.cn

**Keywords:** drug delivery, siRNA therapy, doxorubicin, breast cancer

## Abstract

**Background/Objectives**: Breast cancer remains a leading cause of cancer-related mortality worldwide, primarily due to the systemic toxicity and drug resistance associated with conventional doxorubicin (DOX) therapy. To overcome these limitations, we developed and optimized a novel cationic-ionizable lipid nanoparticle platform, QTPlus, for the co-delivery of DOX and siRNA targeting fibroblast growth factor 2 (siFGF2). **Methods**: The study evaluated the physicochemical properties, cellular uptake, gene regulation, apoptosis induction, and in vivo antitumor efficacy and safety of QTPlus-DOX-siFGF2 in breast cancer models. Results: QTPlus nanoparticles based on the A-066 formulation achieved uniform particle size (~218 nm), low polydispersity (PDI 0.164–0.214), and high encapsulation efficiencies (DOX: 49.56 ± 0.15%; siFGF2: 77.66 ± 1.30%). In vitro release studies revealed a robust pH-responsive profile, characterized by sustained stability at physiological pH (7.4) and rapid burst release at acidic endosomal pH (5.5). In MCF-7 and MDA-MB-231 cells, QTPlus-DOX-siFGF2 significantly enhanced cellular uptake, downregulated FGF2 (0.639-fold) and VIM (0.373-fold), and upregulated CASP3 (3.364-fold in siFGF2 group) and BRCA1 (4.041-fold). Flow cytometry showed markedly increased apoptosis (78.5% vs. 42.65% for QTPlus-DOX alone). In the MDA-MB-231 xenograft model, QTPlus-DOX-siFGF2 achieved 65.87% tumor growth inhibition with stable body weights and favorable trends in cardiotoxic biomarkers. **Conclusions**: These results demonstrate that QTPlus enables effective co-delivery of DOX and siFGF2, producing synergistic antitumor effects through apoptosis induction and suppression of epithelial–mesenchymal transition while improving the safety profile. QTPlus-DOX-siFGF2 represents a promising nanotherapeutic strategy for breast cancer warranting further clinical development.

## 1. Introduction

Breast cancer is the most common malignancy among women and remains a leading cause of cancer-related mortality worldwide. Its incidence has continued to rise in recent years, leading to a major public health challenge. More than 2 million women are diagnosed with breast cancer globally each year, with approximately 500,000 deaths attributed to the disease [1,2]. Conventional treatment methods, including surgery, chemotherapy, and radiotherapy, are often limited by high recurrence rates, drug resistance, and severe side effects [3,4]. Among chemotherapeutic agents, doxorubicin (DOX) is widely used but constrained by nonspecific biodistribution and significant systemic toxicity. With growing insights into the molecular mechanisms underlying tumor microenvironments, targeted therapies have emerged as a promising strategy against breast cancer, offering the potential to enhance efficacy while minimizing side effects through precise modulation of disease-specific molecular pathways [5].

The high expression of FGF2 in breast cancer promotes breast cancer progression through multiple mechanisms, including suppression of apoptosis by increasing survivin expression via an Akt-dependent pathway, which inhibits CASP3 activity and enhances cell survival [6,7]; downregulation of BRCA1 via activation of FGFR2 signaling through the ERK-YY1 axis, leading to reduced BRCA1 transcription and accelerated tumorigenesis, particularly in triple-negative breast cancer [8]; and facilitation of epithelial–mesenchymal transition (EMT) by activating FGFR1 paracrine loops in the tumor stroma, resulting in increased VIM expression that supports cell migration and invasion [9]. Inhibiting the expression of FGF2 can effectively suppress tumor growth and metastasis, and has been shown to enhance the efficacy of doxorubicin in breast cancer treatment [10]. For instance, Hu Y et al. and Li et al. introduced that high FGF2 expression correlates with doxorubicin resistance by promoting nuclear translocation of FGF2, which upregulates DNA-dependent protein kinase (DNA-PK) expression and accelerates DNA repair, thereby reducing doxorubicin-induced apoptosis [11]; conversely, inhibiting FGF2 (e.g., via miR-205 targeting) downregulates PI3K/AKT signaling, restores chemosensitivity, and increases apoptosis in doxorubicin-resistant cells [12]. This evidence suggested that FGF2 inhibition could serve as a promising therapeutic strategy in combination with doxorubicin against breast cancer.

Small interfering RNA (siRNA) is a powerful tool for gene silencing, capable of inhibiting gene expression by specifically binding to target mRNA and inducing nuclease degradation. However, siRNA faces challenges such as stability, selectivity, and pharmacokinetics when delivered in vivo, necessitating the development of effective delivery systems to enhance its therapeutic effects [13]. In recent years, nanotechnology has made significant progress in the field of drug delivery, with nanocarriers being an ideal choice for siRNA delivery due to their unique physicochemical properties, such as small size, high specific surface area, and good biocompatibility [14].

In recent years, ionizable lipid nanoparticles (LNPs) have emerged as a promising platform for siRNA delivery due to their ability to encapsulate nucleic acids efficiently and facilitate endosomal escape through pH-dependent charge modulation [15]. Recent advances in LNP-based siRNA technologies have demonstrated substantial progress in cancer treatment, including improved tumor accumulation and gene silencing in solid tumors [16,17]. However, most established LNP systems, such as those based on MC3 (used in Onpattro^®^) or SM-102 (used in mRNA COVID-19 vaccines), are primarily optimized for hepatic delivery or mRNA vaccination and often exhibit limited efficiency in solid tumors due to predominant liver tropism and suboptimal co-delivery of small-molecule drugs [18]. In contrast, the QTPlus platform employs a unique combination of a quaternary amine-based cationic lipid (permanent positive charge) and a tertiary amine-based ionizable lipid with minimal PEG-lipid content (1.2 mol%), enabling balanced co-encapsulation and cytosolic delivery of both anionic siRNA and hydrophobic chemotherapeutic agents while improving extravasation into solid tumors.

In our previous research, we have developed a novel lipid nanoparticle platform QTsome, using a combination of a quaternary amine-based cationic lipid and a tertiary amine-based ionizable lipid to enhance nucleic acid delivery. The composition of QTsome was further optimized into QTPlus based on the molar ratio published by Moderna, Pfizer/BioNTech, and Alnylam Pharmaceuticals, which utilized a minimal amount of PEG lipids and an optimal molar ratio between PEG lipids, cationic lipids, and ionizable lipids [19]. We demonstrated that QTPlus successfully delivered antisense oligonucleotide targeting microRNA-21 and inhibited non-small cell lung cancer growth in vitro and in vivo [20,21]. However, whether this formulation and selected functional lipids are also suitable for siRNA delivery against breast cancer remains unclear.

We hypothesized that QTPlus-mediated co-delivery of siFGF2 and DOX would produce synergistic antitumor effects by simultaneously silencing FGF2 (thereby suppressing EMT, restoring chemosensitivity, and promoting apoptosis) and exerting direct chemotherapeutic cytotoxicity, while the nanoparticle encapsulation would reduce systemic toxicity compared with free DOX.

In this study, we screened different ionizable lipids in combination with cationic lipids in QTPlus for the single delivery of siRNA targeting FGF2 (siFGF2) and the combinational delivery of both siFGF2 and doxorubicin against breast cancer. Based on the comprehensive experimental results, this study evaluates the performance of various ionizable lipid formulations for the co-delivery of siFGF2 and doxorubicin (DOX) using the QTPlus platform. Characterization studies revealed that the A-066 lipid formulation exhibited the optimal balance of particle size, zeta potential, and encapsulation efficiency, achieving the highest DOX encapsulation (49.56 ± 0.15%) while maintaining competitive siFGF2 encapsulation (77.66 ± 1.30%), establishing it as the superior choice for dual-drug delivery. Moreover, the formulation exhibited a distinct pH-responsive release behavior, which prevents premature drug leakage in circulation while facilitating rapid payload discharge under acidic conditions. Cellular uptake experiments further confirmed A-066 as the most effective lipid for balanced internalization of both therapeutic agents, enhancing DOX delivery without compromising siFGF2 uptake. RT-qPCR analysis demonstrated significant downregulation of FGF2 and VIM, alongside upregulation of apoptosis marker CASP3 and BRCA1, particularly in the QTPlus-DOX-siFGF2 formulation, indicating robust therapeutic potential. In vivo studies using a subcutaneous xenograft model validated these findings, with the QTPlus-DOX-siFGF2 formulation achieving the most significant tumor growth suppression (TGI% of 65.87%) and minimal systemic toxicity, as evidenced by stable body weights. These results collectively highlight the potential of A-066-based QTPlus-DOX-siFGF2 as a promising nanotherapeutic platform for synergistic cancer treatment, warranting further investigation into its clinical applicability.

## 2. Materials and Methods

### 2.1. Materials

Cholesterol, 1-(2,3-bis(((9Z,12Z)-octadeca-9,12-dien-1-yl)oxy)propyl)pyrrolidine (A-066), 1,2-Distearoyl-sn-glycerol-3-phosphocholine (DSPC), 1,2-Dimyristoyl-rac-glycero-3-methoxypolyethylene glycol-2000 (DMG-PEG 2000), 1,2-Dioleoyl-3-trimethylammonium-propane (DOTAP), 1,2-Dioleoyl-3-dimethylaminopropane, (DODMA), Heptadecane-9-yl 8-((2-hydroxyethyl)(6-oxo-6-(undecyloxy)hexyl)amino)octanoate (SM-102), 1-[2,3-Bis [(9Z,12Z)-octadeca-9,12-dien-1-yloxy]propyl]pyrrolidine (A-066), (6Z,9Z,28Z,31Z)-Heptatriaconta-6,9,28,31-tetraen-19-yl 4-(dimethylamino)butanoate (DLin-MC3-DMA, MC3), [(4-Hydroxybutyl)imino]di-6,1-hexanediyl bis(2-hexyldecanoate) (ALC-0315), ethanol, Sodium acetate, Doxorubicin hydrochloride (DOX).

siRNA sequences targeting human FGF2 were designed as follow: Sense strand: 5′-uauacugcccaguucguuucaguGC-3′; Antisense strand: 5′-gcacugaaacgaacugggcaguaua*a*a-3′(Capital represents DNA molecule, and “*” represents phosphorothioated backbone). The siRNA sequences targeting human FGF2 were designed with phosphorothioate (*) modifications at the 3′ end of the antisense strand to enhance nuclease resistance and stability during delivery, while minimizing off-target effects and immunogenicity, as supported by prior siRNA design strategies [22].

### 2.2. Methods

#### 2.2.1. Preparation of Empty QTPlus and DOX-Encapsulating QTPlus (eQTPlus and QTPlus-DOX)

The eQTPlus was prepared by hand, involving rapid injection of the lipid mixture into the acetic acid buffer. Detailed compositions of QTPlus can be found in Table 1. The sample of QTPlus-DOX, DOTAP, A-066, DSPC, Cholesterol and DMG-PEG 2000 each weighed at 50 mg and dissolved in 1 mL of anhydrous ethanol to obtain stock solutions. The lipids were then mixed at a molar ratio of 1/40/5.6/25.6/1.2 and vortexed in an Eppendorf tube. The lipid mixture and DOX stock solutions were mixed, and 200 μL of the resulting mixture was then injected into 500 μL of acetate buffer (50 mM, pH 5.2) under constant vortexing, followed by probe sonication for 90 s on ice using an ultrasonic cell disruptor (XIUILAB XU-JY96-IIN, Shanghai, China). The resulting eQTPlus and QTPlus-DOX were stored at 4 °C until further use and briefly re-sonicated prior to siFGF2 encapsulation to ensure uniform particle size. Free DOX was not removed after preparation for in vitro and animal experiments, so cells were exposed to the total (encapsulated + free) DOX content.

#### 2.2.2. Preparation of eQTPlus/QTPlus-DOX Encapsulating siFGF2 (QTPlussiFGF2/QTPlus-DOX-siFGF2)

siFGF2 solutions were prepared in DEPC-treated water (KeyGEN Biotech Co., Nanjing, China). siFGF2 solutions were mixed with eQTPlus or QTPlus-DOX under constant vortex to generate QTPlus-siFGF2 or QTPlus-DOX-siFGF2 with a final lipid-to-siRNA at 20/1 (*w*/*w*).

#### 2.2.3. Characterization of QTPlus-siFGF2 and QTPlus-DOX-siFGF2

The mean hydrodynamic particle diameters, zeta-potential (ζ) and polydispersity index (PDI) of the QTPlus-siFGF2 and QTPlus-DOX-siFGF2 were measured using dynamic light scattering (DLS) using a NanoBrook Omni (Brookhaven, Brookhaven, GA, USA). To measure the particle size and PDI, QTPlus samples were diluted 20-fold with deionized water. For zeta potential measurement, QTPlus samples were diluted 50-fold with deionized water. The encapsulation efficiency of DOX in QTPlus was measured in triplicate by UV spectrophotometry (Pono-500, PORABIO, Hangzhou, China). A linear standard curve (y = 0.0468x + 0.0091, R^2^ = 0.9999) was established over a concentration range of 0.1–10 mg/mL. The encapsulation efficiency of siFGF2 in QTPlus was determined using the RiboGreen RNA Quantitation Kit (Beyotime Biotech Inc., Shanghai, China). Fluorescence intensity was measured using a microplate reader with excitation and emission wavelengths set at 485 nm and 528 nm, respectively (Tristar 5, Berthold, Bad Wildbad, Germany). A linear standard curve (y = 0.4299x + 2.3192, R^2^ = 0.9988) generated using siRNA concentrations of 0–200 nM.

The encapsulation efficiency (EE, %) was calculated based on the difference between the total siRNA concentration and the free siRNA concentration, as follows:

$$EE,\%=\frac{Total\;siRNA\;concentration-Free\;siRNA\;concentration}{Total\;siRNA\;concentration}\times 100$$

### 2.3. In Vitro Drug Release Study

The in vitro release profiles of DOX and siFGF2 from QTPlus formulations were evaluated using a dialysis method. Briefly, free DOX, free siFGF2, QTPlus-DOX, QTPlus-siFGF2, and QTPlus-DOX-siFGF2 formulations were separately transferred into dialysis bags (molecular weight cut-off, MWCO: 100 kDa). Each dialysis bag containing 1 mL of sample was immersed in 20 mL of release medium. Phosphate-buffered saline (PBS) at pH 7.4 and pH 5.5 was used to simulate physiological conditions and the acidic tumor microenvironment/endosomal conditions, respectively. The release study was conducted at 37 ± 0.5 °C under gentle shaking at 100 rpm.

At predetermined time intervals (0.5, 1, 2, 4, 8, 12, 24 h), release medium was withdrawn and replaced with an equal volume of fresh prewarmed PBS to maintain sink conditions. The amount of released DOX was quantified using Nanodrop spectrophotometer (Pono-500, PORABIO, China) at 480 nm. For siFGF2, the released RNA was quantified using the RiboGreen RNA assay kit (Beyotime Biotech Inc., China) according to the manufacturer’s instructions. The cumulative release percentage (CR, %) was calculated according to the following equation:

$$CR,\%=\frac{V_{e}\times \sum_{i=1}^{n-1}C_{i}+V_{0}\times C_{n}}{W_{total}}\times 100$$

where V_e_ is the volume of each sample taken (unit: mL), the volume of the sample aspirated from the release medium at each preset time point, ensuring that the volume of each sample taken is consistent; C_i_ is the concentration of the drug in the release medium at the i-th sampling (unit: μg/mL or mg/mL), quantitatively determined by detection methods such as UV-Vis spectrophotometry or fluorescence spectroscopy; *n* is the total number of samplings, corresponding to the preset release time points (e.g., 0.5, 1, 2, 4, 8, 12, 24 h); V_0_ is the initial total volume of the release medium (unit: mL), which is the total volume of the release system outside the dialysis bag; C_n_ is the concentration of the drug in the release medium at the last (*n*-th) sampling (unit: μg/mL or mg/mL); and W_total_ is the total encapsulated amount of the drug in the nanocarriers (unit: μg or mg), the total mass of the drug initially encapsulated in the nanoparticles, obtained from the encapsulation efficiency determination experiment. All experiments were performed in triplicate.

### 2.4. Cell Culture

MCF-7 and MDA-MB-231 cell lines were purchased from KeyGEN BioTECH Co. All cell lines were cultured in DMEM medium supplemented with 10% fetal bovine serum. Cells were maintained at 37 °C and grown under a humidified atmosphere containing 5% CO_2_.

### 2.5. Cellular Uptake Assay

To evaluate the uptake efficiency of DOX and siFGF2 by QTPlus in the breast cancer cells, MCF-7 cells were seeded at 5000 cells per well in 96-well plates 24 h before treatments. QTPlus were prepared using siFGF2 labeled with Cy5 fluorescent dye. To trace lipid-based nanoparticles, DiO was incorporated into the lipid mixture (at 1% (*v*/*v*) of total lipids) during the ethanol phase preparation before rapid injection into the buffer, ensuring it was embedded in the lipid bilayer. Fluorescence detection was performed using a microplate reader with the following excitation and emission wavelengths: 484 nm/524 nm for DiO, 480 nm/600 nm for DOX, and 650 nm/690 nm for siRNA. Cells were treated with QTPlus previously described (DOX at 5 nM concentrations, siFGF2 at 100 nM). After 48 h treatment, cells were washed three times with PBS and the fluorescence intensity was measured using a microplate reader (Infinite M Nano, Jiangsu Overseas Group International Trade & Cooperation, Nanjing, China).

### 2.6. In Vitro Gene Regulation Studies

MCF-7 and MDA-MB-231 cells were seeded at 3 × 10^5^ cells per well in 6-well plates 24 h before treatments. Cells were treated with QTPlus (DOX at 13μM concentrations, siFGF2 at 200 nM concentrations). Total RNA was extracted from untreated and all treated groups 24 h after treatments using the total RNA extraction reagent (KeyGEN BioTECH Co., China) method, and the purity and integrity of RNAs were tested by measuring A260/A280.

After synthesizing cDNA using the BioRT High-Sensitivity cDNA First-Strand Synthesis Kit for RT-PCR (Vazyme R333-C1, Nanjing, China), real-time qPCR (RT-qPCR) was carried out using SYBR green master mix (BioEasy BSB113S1, Shenzhen, China) and the 96 A instrument (Bioer Technology, Hangzhou, China). The forward and reverse primer sequences for Gapdh, FGF2, CASP3, BRCA1 and VIM were listed in Table S1. Relative expression of target mRNA was normalized to Gapdh. The ΔΔCT method was used to measure fold increase in genes in comparison to the control group.

### 2.7. Cellular Flow Cytometry

The apoptosis in MCF-7 cells after treatment with the prepared QTPlus was evaluated using an Annexin V-FITC/PI apoptosis detection kit (KeyGEN BioTECH Co., China). Cells were seeded at a density of 3× 10^5^ per well in 6-well plates 24 h before treatments. The cells were then treated with QTPlus previously described (DOX at 13 μM concentrations, siFGF2 at 200 nM). After 24 h treatment, the cells were rinsed with PBS before being collected and resuspended in 300 μL of binding buffer. Cells were stained with Annexin V-FITC/PI following the manufacturer’s protocol. The fluorescence intensity of the stained cells was then analyzed by flow cytometry (BECKMAN COULTER CytoFLEX, Indianapolis, IN, USA), and Annexin V/PI staining enabled discrimination of live (Annexin V^−^/PI^−^), early-apoptotic (Annexin V^+^/PI^−^), late-apoptotic (Annexin V^+^/PI^+^), and necrotic (Annexin V^−^/PI^+^) populations [23]. The 13 μM DOX concentration was selected for the 24 h apoptosis assay because it approximates peak intratumoral DOX levels achievable after systemic administration (up to 50-fold higher than plasma) and allows reliable detection of early apoptotic events within a short incubation period, consistent with prior breast cancer studies [24,25]. In contrast, the 72 h CCK-8 assay used lower concentrations (nM to μM range) to determine long-term IC50 values.

### 2.8. CCK-8 Assay

MCF-7 cells and MDA-MB-231 cells were seeded at 5000 cells per well in 96-well plates 24 h before treatments. Cells were treated with Untreated, free DOX, QTPlus-DOX and QTPlus-DOX-siFGF2, (DOX concentrations from 0 nM to 300 nM or from 0 μM to 300 μM). After 72 h treatment, cell viability was examined by CCK-8 assay (KeyGEN BioTECH Co., China). The synergistic effects of DOX and siFGF2 were determined by GraphPad Prism.

### 2.9. In Vivo Antitumor Efficacy Study

To study the antitumor activity of siFGF2 and DOX, the MDA-MB-231 xenograft mouse model was generated by subcutaneously inoculating nude mice with 2.5 × 10^6^ cells per mouse on the right flank. The mice were Balb/c nude mice, obtained from Spefu (Suzhou) Biotechnology Co., Ltd. (Suzhou, China). The animals were 5 weeks old, female, and sourced under the Experimental Animal Production License (SCXK 2022-0006, Certificate No. A202505160430) and used in accordance with the Experimental Animal Use License (SYXK 2023-0001). This study was approved by the Ethics Committee of Nanjing Advanced Academy of Life and Health (Animal Welfare Assurance Number: NAALH-W-2504030). Institutional Animal Care and Use Committee (IACUC) (Ethical Approval No. NAALH-W-2504030). Treatments were initiated once tumors reached to volumes of 80–100 mm^3^, animals were randomized into groups. Mice were block-randomized into treatment groups by an independent researcher prior to the first dose using a computer-generated randomization schedule. Tumor volume measurements and body weight assessments were performed by investigators blinded to treatment allocation to minimize observer bias. Mice (*n* = 6 or =12) were intravenously injected with saline, free DOX (1 mg/kg), QTPlus-siFGF2 (0.6 mg/kg and 1.2 mg/kg) and QTPlus-DOX-siFGF2 (1 mg/kg DOX, 0.6 mg/kg siFGF2). A DOX dose of 1 mg/kg was selected to minimize toxicity while evaluating synergy, as higher doses (e.g., 5–10 mg/kg) often cause severe cardiotoxicity in nude mice [24]. The administered DOX dose (1 mg/kg) was calculated based on the total (encapsulated + free) DOX content measured post-preparation. This aligns with prior studies optimizing for safety in combination therapies [26]. All mice were dosed every 3 days for 5 doses. Tumor growth and body weight were monitored, and the tumor volumes were calculated according to the formula:

$$Tumor\;Volume=\frac{Length\times {Width}^{2}}{2}$$

All mice were euthanized one day after the last dose. TGI% was determined by the formula:

$$TGI,\%=\frac{1-(T_{t}/T_{0})/(C_{t}/C_{0})}{1-C_{0}/C_{t}}\times 100$$

where T_t_ stands for the average tumor volume of the treatment group on the day of measurement, T_0_ stands for the average tumor volume of the treatment group at day 0, C_t_ stands for the average tumor volume of the control group on the day of measurement, and C_0_ stands for the average tumor volume of the control group at day 0. TGI% > 50% was considered meaningful. The treatment groups consisted of *n* = 6 mice per group, a sample size predetermined to provide sufficient statistical power (α = 0.05) for evaluating tumor growth inhibition. The saline and free DOX control groups included *n* = 12 mice per group to establish a more robust baseline for natural tumor growth and systemic toxicity. All animals (*n* = 12 for saline/free DOX and *n* = 6 for formulations) were continuously monitored for tumor volume and body weight, and all respective data points were fully incorporated into the final statistical analyses. The statistical methodologies employed (e.g., one-way ANOVA or mixed-effects models) naturally accommodate these unequal sample sizes, ensuring valid comparisons across all groups.

### 2.10. Hematoxylin and Eosin (H&E) Staining

Tissues, including the heart, liver, spleen, lung, kidney, and tumor, were collected and fixed in 10% neutral buffered formalin for histopathological examination. Samples were obtained from five experimental groups: (1) saline, (2) free DOX (1 mg/kg), (3) QTPlus-siFGF2 (0.6 mg/kg), (4) QTPlus-siFGF2 (1.2 mg/kg), and (5) QTPlus-DOX- siFGF2 (1 mg/kg DOX, 0.6 mg/kg siFGF2). Fixed tissues were embedded in paraffin and sectioned at 4 μm thickness. Sections were deparaffinized in xylene for 5 min (twice), rehydrated through a descending ethanol series (100%, 95%, 85%, 70%, 5 min each), and rinsed in PBS three times for 3 min each. H&E staining was conducted using a commercial staining kit (KGA224, Jiangsu KGI Biological Technology Co., Ltd., Nanjing, China). Nuclei were stained by immersing slides in hematoxylin for 3–5 min, followed by water rinsing for 30–60 s. Differentiation was performed in differentiation solution I for 20 s, rinsed, followed by immersion in differentiation solution II for 40 s and a water rinse. Cytoplasmic staining was performed using eosin for 2 min. Sections were then rinsed twice with the color enhancement solution, air-dried, and mounted using neutral balsam (Sinopharm Chemical Reagent Co., Ltd., Shanghai, China). Histological observation was performed under a biological upright microscope (BX63, Olympus, Tokyo, Japan).

### 2.11. Biochemistry Parameters

For blood biochemical assessments, samples were collected via retro-orbital plexus under light anesthesia. Serum samples were allowed to clot at room temperature for 2 h or kept overnight at 4 °C, except for glucose measurement, which required immediate processing. After coagulation, samples were centrifuged at 121× *g* for 15 min at 2–8 °C (SLX-1024F, Wuhan Servicebio Technology Co., Ltd., Wuhan, China). The supernatant was either used immediately for analysis or aliquoted and stored at −20 °C or −80 °C. Repeated freeze–thaw cycles were strictly avoided. Before testing, frozen samples were thawed and recentrifuged to remove any precipitates. For plasma collection, blood samples were collected in heparinized tubes and centrifuged at 121× *g* for 15 min at 2–8 °C (SLX-1024F, Wuhan Servicebio Technology Co., Ltd., Wuhan, China) within 30 min of collection. The clarified plasma samples were either analyzed immediately or stored at −20 °C or −80 °C in aliquots. All samples used for biochemical analysis were required to be clear and non-hemolyzed. Quantitative detection of serum biomarkers was carried out using a microplate reader (BioTek Epoch, Winooski, VT, USA) or an automated biochemical analyzer (Chemray 240, Mindray, Shenzhen, China) following the manufacturer’s instructions.

### 2.12. Statistical Analysis

All studies were performed in triplicate unless otherwise specified. Data are presented as means ± standard deviations unless otherwise indicated. Data normality was assessed using the Shapiro–Wilk test and homogeneity of variance by Levene’s test. Two-way or one-way ANOVA followed by Tukey’s honestly significant difference (HSD) post hoc test was used for multiple-group comparisons; unpaired Student’s *t*-test was used for two-group comparisons (GraphPad Prism 10.0). Batch-to-batch variability of QTPlus formulations was monitored across at least three independent batches and remained consistent for key parameters (particle size, PDI, and encapsulation efficiency).

## 3. Results

### 3.1. Characterization of QTPlus

**Figure 1:**
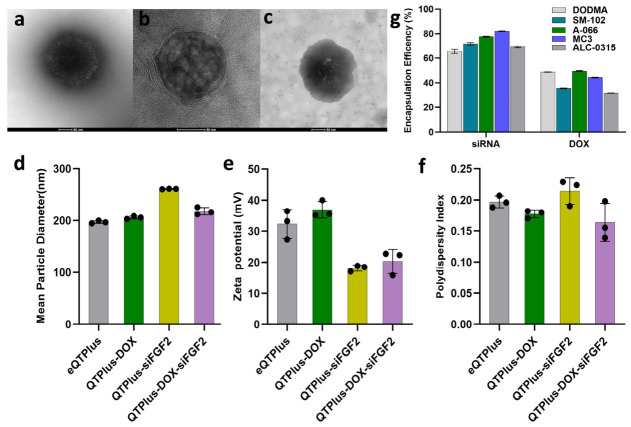
Morphological and physicochemical characterization of liposomal formulations. (**a**–**c**) Representative TEM images of QTPlus-DOX, QTPlus-siFGF2, and QTPlus-DOX-siFGF2. All formulations exhibited spherical morphology with uniform size distribution. Scale bar: 50 nm. (**d**) Hydrodynamic diameter, (**e**) zeta potential, and (**f**) polydispersity index (PDI) of eQTPlus, QTPlus-DOX, QTPlus-siFGF2, and QTPlus-DOX-siFGF2. (**g**) Encapsulation efficiency of DOX and siFGF2 for different ionizable lipids, with A-066 identified as the preferred formulation. Data represent mean ± SD from three independent measurements.

In Figure 1a–c, all formulations displayed well-defined spherical nanostructures with characteristic bilayer membranes. The absence of particle aggregation or structural collapse suggests successful stabilization during preparation. Notably, the dual-loaded QTPlus-DOX-siFGF2 (Figure 1c, inset) maintained a compact spherical shape with clearly visible bilayer boundaries, indicating that QTPlus-DOX-siFGF2 did not compromise the liposomal architecture. The preserved bilayer structure across all formulations supports their potential for controlled drug release and biological stability.

In Figure 1d, the mean particle size of the eQTPlus was 197.02 ± 2.15 nm. QTPlus-DOX resulted in a slight, non-significant increase in size to 205.88 ± 2.31 nm. QTPlus-siFGF2 exhibited a significantly larger mean diameter of 261.02 ± 0.46 nm compared to eQTPlus. QTPlus-DOX-siFGF2 demonstrated an intermediate particle size of 218.18 ± 5.74 nm, which was significantly larger than eQTPlus (21.17 nm increase) and QTPlus-DOX (12.30 nm increase), but smaller than QTPlus-siFGF2 (43.02 nm reduction). Zeta potential measurements (Figure 1e) revealed a moderately positive surface charge for eQTPlus (+32.35 ± 3.77 mV). Incorporation of QTPlus-DOX significantly increased the positive charge from 32.35 ± 3.77 mV to 36.93 ± 2.19 mV. In contrast, QTPlus-siFGF2 significantly reduced the zeta potential to +18.14 ± 0.77 mV. No significant differences in zeta potential were observed between QTPlus-siFGF2 and QTPlus-DOX-siFGF2, suggesting that loading of DOX did not change the electrostatic interaction between QTPlus and siFGF2. All formulations displayed excellent colloidal stability and homogeneity, with PDI values in the range of 0.164 to 0.214 (Figure 1f). The morphological features of eQTPlus, QTPlus-DOX, QTPlus-siFGF2, and QTPlus-DOX-siFGF2 were analyzed by TEM to validate their structural integrity. Importantly, the co-injection preparation process demonstrated excellent reproducibility. Across at least three independent batches, the QTPlus formulations exhibited minimal batch-to-batch variability in key physicochemical parameters, maintaining highly consistent particle sizes, PDIs, and encapsulation efficiencies.

The encapsulation efficiency (EE%) of both DOX and siFGF2 was systematically evaluated across five ionizable lipid formulations (Figure 1g). Among the tested lipids, A-066 demonstrated superior performance for DOX encapsulation with an EE% of 49.56 ± 0.15%, the highest value observed. This represented a 1.7% increase over DODMA (48.73 ± 0.22%), 11.5% increase over SM-102 (35.33 ± 0.31%), 10.6% increase over D-Lin-MC3-DMA (44.30 ± 0.21%), and 35.7% increase over ALC-0315 (31.84 ± 0.08%). For siFGF2 encapsulation, D-Lin-MC3-DMA showed the highest efficiency at 82.08 ± 0.22%, followed by A-066 (77.66 ± 1.30%), SM-102 (71.81 ± 0.28%), ALC-0315 (69.12 ± 0.61%), and DODMA (65.50 ± 0.82%). Notably, A-066 maintained competitive siRNA encapsulation while achieving the optimal DOX loading. This balanced co-encapsulation profile, combined with its leading DOX EE%, established A-066 as the optimal ionizable lipid for subsequent studies.

### 3.2. In Vitro Drug Release Study of DOX and siFGF2

**Figure 2:**
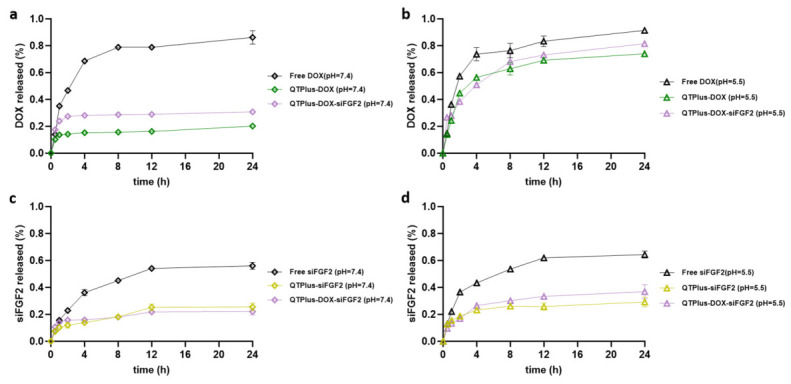
In vitro cumulative release profiles of DOX and siFGF2. Cumulative release of DOX from Free DOX, QTPlus-DOX, and QTPlus-DOX-siFGF2 formulations at (**a**) pH 7.4 and (**b**) pH 5.5. Cumulative release of siFGF2 from Free siFGF2, QTPlus-siFGF2, and QTPlus-DOX-siFGF2 formulations at (**c**) pH 7.4 and (**d**) pH 5.5. Data are presented as mean ± SD (*n* = 3).

To evaluate the pH-responsive release behavior and the influence of co-encapsulation, the in vitro release profiles of DOX and siFGF2 were evaluated at pH 7.4 (physiological conditions) and pH 5.5 (endosomal/tumor microenvironment conditions). As shown in Figure 2a,b, free DOX exhibited rapid diffusion with cumulative release reaching 86.18% and 91.47% at pH 7.4 and 5.5 over 24 h, respectively. At pH 7.4, the release of DOX from QTPlus formulations was significantly restricted, with 20.19% and 30.75% released from QTPlus-DOX and QTPlus-DOX-siFGF2, respectively, indicating high stability in systemic circulation. Conversely, at pH 5.5, a rapid burst release of DOX was triggered, reaching 74.07% for QTPlus-DOX and 81.58% for the dual-loaded QTPlus-DOX-siFGF2 (Figure S1a,b).

For siFGF2 (Figure 2c,d), free siFGF2 diffused reasonably well, achieving 56.05% and 64.35% release at pH 7.4 and 5.5 over 24 h. When formulated in QTPlus, the release of siFGF2 at pH 7.4 was well-controlled at 25.55% (QTPlus-siFGF2) and 22.05% (QTPlus-DOX-siFGF2). At pH 5.5, the cumulative release slightly increased to 29.18% and 36.92%, respectively (Figure S1c,d). The synchronous encapsulation of DOX did not hinder the release characteristics of siFGF2.

### 3.3. Cellular Uptake Studies

**Figure 3:**
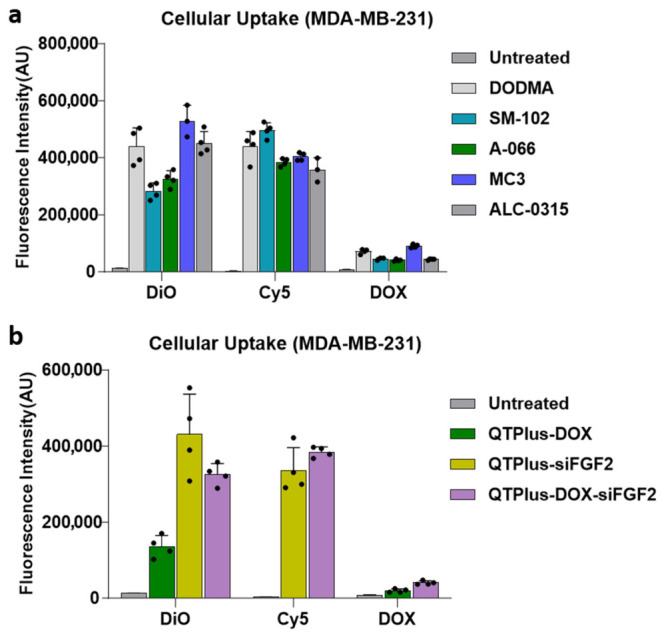
Quantitative analysis of cellular internalization components in MDA-MB-231 cells after treatment with QTPlus-siFGF2, QTPlus-DOX, and QTPlus-DOX-siFGF2. (**a**) Fluorescence indicating the cellular uptake of QTPlus formulated with five cationic lipids (DODMA, SM-102, A-066, MC3, and ALC-0315). (**b**) Intrinsic fluorescence formulations based on A-066 (QTPlus-siFGF2, QTPlus-DOX, and QTPlus-DOX-siFGF2). Data represent mean ± SD (*n* = 4).

To assess the impact of different ionizable lipids on QTPlus-DOX-siFGF2 performance, we compared five formulations differing only in their ionizable lipid: DODMA, SM-102, A-066, MC3, and ALC-0315. As shown in Figure 3a, the DiO fluorescent signals indicated that ALC-0315 and MC3 showed the highest uptake, followed by DODMA, with SM-102 and A-066 exhibiting lower uptake. The Cy5 signals for siFGF2 uptake revealed that DODMA and SM-102 had the highest levels, while MC3 and A-066 showed comparable uptake and ALC-0315 showed the lowest level. DOX fluorescence was highest in DODMA and MC3, followed by SM-102, with A-066 and ALC-0315 showing lower levels. Quantitative assessment of nanoparticle internalization revealed distinct formulation-dependent uptake profiles after 48 h incubation (Figure 3b). QTPlus-siFGF2 and QTPlus-DOX-siFGF2 exhibited significantly enhanced uptake compared to QTPlus-DOX based on DiO signal. Cy5 signals indicated comparable cellular uptake of siFGF2 between QTPlus-DOX-siFGF2 and QTPlus-siFGF2 formulations. Fluorescent signals of DOX showed higher delivery efficiency in QTPlus-DOX-siFGF2 compared with QTPlus-DOX.

### 3.4. RT-qPCR Validation of Gene Regulation

**Figure 4:**
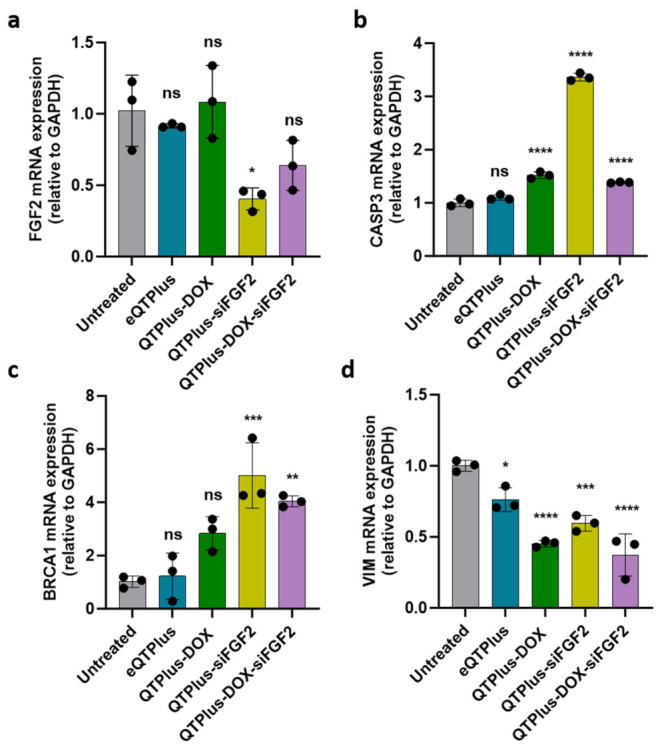
RT-qPCR results from MDA-MB-231 cells treated with QTPlus-DOX, QTPlus-siFGF2, and QTPlus-DOX-siFGF2. mRNA expressions of (**a**) FGF2, (**b**) CASP3, (**c**) BRCA1, and (**d**) VIM were detected. Data represent mean ± SD of relative expression (*n* = 3). * *p* < 0.05, ** *p* < 0.01, *** *p* < 0.001, **** *p* < 0.0001, ns, no significant difference vs. untreated by Tukey’s HSD test after one-way ANOVA.

As shown in Figure 4, treatment with QTPlus-siFGF2 and QTPlus-DOX-siFGF2 resulted in downregulation of FGF2 andVIMVIM, and upregulation of CASP3CASP3and BRCA1. RT-qPCR analysis revealed distinct gene expression patterns across different treatment groups. Significant downregulation of FGF2 was observed in cells treated with both QTPlus-siFGF2 (0.404 ± 0.077-fold) and QTPlus-DOX-siFGF2 (0.639 ± 0.175-fold) compared with QTPlus-DOX (1.085 ± 0.254-fold) or eQTPlus. For the apoptosis marker CASP3, QTPlus-siFGF2 treatment induced significant upregulation (3.364 ± 0.071-fold). Substantial BRCA1 upregulation was detected in drug-loading groups, with the highest upregulation in the QTPlus-siFGF2 formulation (5.009 ± 1.228-fold) and QTPlus-DOX-siFGF2 formulation (4.041 ± 0.211-fold). The epithelial–mesenchymal transition marker VIM was markedly downregulated by QTPlus-DOX (0.453 ± 0.022-fold), QTPlus-siFGF2 (0.596 ± 0.056-fold) and QTPlus-DOX-siFGF2 (0.373 ± 0.1149-fold) formulations.

### 3.5. Flow Cytometry Analysis of Apoptosis

**Figure 5:**
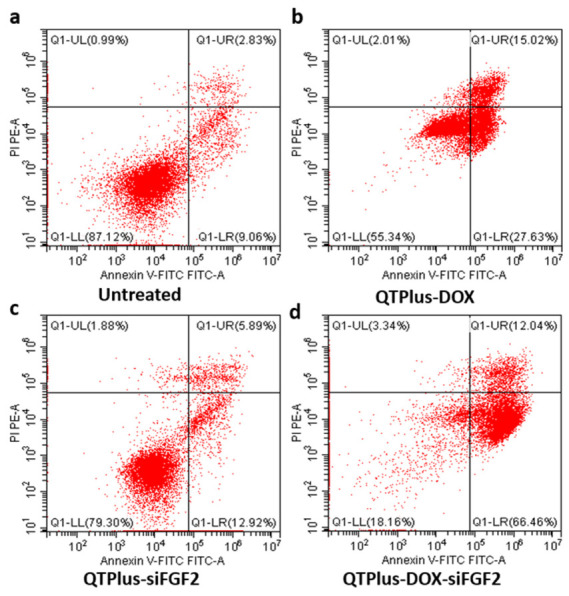
Flow cytometry analysis of cell apoptosis in different treatment groups. The table presents the percentages of cells in each quadrant: UL (necrotic cells), UR (late apoptotic cells), LL (viable cells), LR (early apoptotic cells), and the total apoptosis percentage for (**a**) the Untreated, (**b**) QTPlus-DOX, (**c**) QTPlus-siFGF2, and (**d**) QTPlus-DOX-siFGF2 groups.

As shown in Figure 5, the total apoptotic rate increased from 11.89% in the untreated group to 42.65% with QTPlus-DOX and 78.5% with QTPlus-DOX-siFGF2. Cell apoptosis was evaluated using flow cytometry with Annexin V-FITC and propidium iodide (PI) staining to differentiate between viable, early apoptotic, late apoptotic, and necrotic cells. In the Untreated group, the majority of cells were viable (87.12% in the lower left quadrant), resulting in a total apoptotic rate of 11.89%. Treatment with QTPlus-DOX significantly increased the total apoptotic rate to 42.65%. The QTPlus-siFGF2 group showed a moderate increase in apoptosis to 18.81%. Notably, the QTPlus-DOX-siFGF2 group exhibited the highest apoptotic rate of 78.5%, primarily due to a substantial increase in early apoptotic cells (66.46%).

### 3.6. In Vitro Cytotoxicity

**Figure 6:**
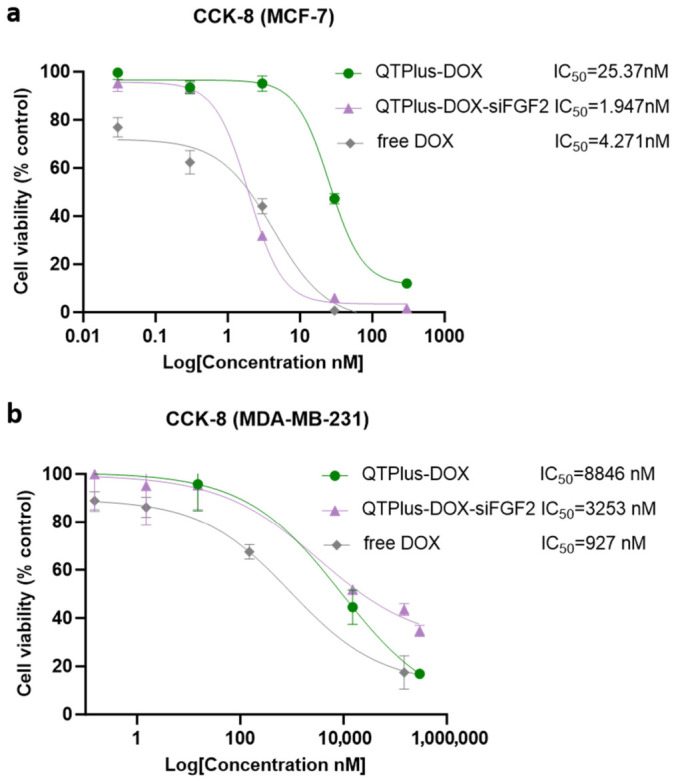
Cytotoxicity of free DOX, QTPlus-DOX, and QTPlus-DOX-siFGF2 on (**a**) MCF-7 and (**b**) MDA-MB-231 cells. Cells were treated with various concentrations (0 nM to 300 nM or 0 μM to 300 μM) of DOX for 72 h. Cell viability was determined using the CCK-8 assay. IC_50_ values were calculated using GraphPad Prism. Data are presented as mean ± SD (*n* = 3).

As shown in Figure 6, free DOX exhibited the lowest IC_50_ in MDA-MB-231 cells (927 nM), followed by QTPlus-DOX-siFGF2 (3253 nM) and QTPlus-DOX (8846 nM). To evaluate the in vitro cytotoxicity of different formulations, CCK-8 assays were performed on MCF-7 and MDA-MB-231 cells after treatment with free DOX, QTPlus-DOX, and QTPlus-DOX-siFGF2. In MCF-7 cells, IC_50_ values were 4.271 nM for free DOX, 25.37 nM for QTPlus-DOX, and 1.947 nM for QTPlus-DOX-siFGF2. In MDA-MB-231 cells, IC_50_ values were 927 nM for free DOX, 8846 nM for QTPlus-DOX, and 3253 nM for QTPlus-DOX-siFGF2. The dose–response curves are presented in Figure 6. The further reduction in cytotoxicity observed with QTPlus-DOX-siFGF2 suggests that the incorporation of siFGF2 enhances the cytotoxicity of DOX through the regulation of FGF2-related signaling pathways, as well as other downstream pathways known to modulate DOX efficacy. This includes pathways the p53 and PI3K/Akt pathways, which have been shown to modulate DOX-induced cell death and chemoresistance [27,28].

### 3.7. Evaluating the In Vivo Performance of QTPlus-DOX-siFGF2

**Figure 7:**
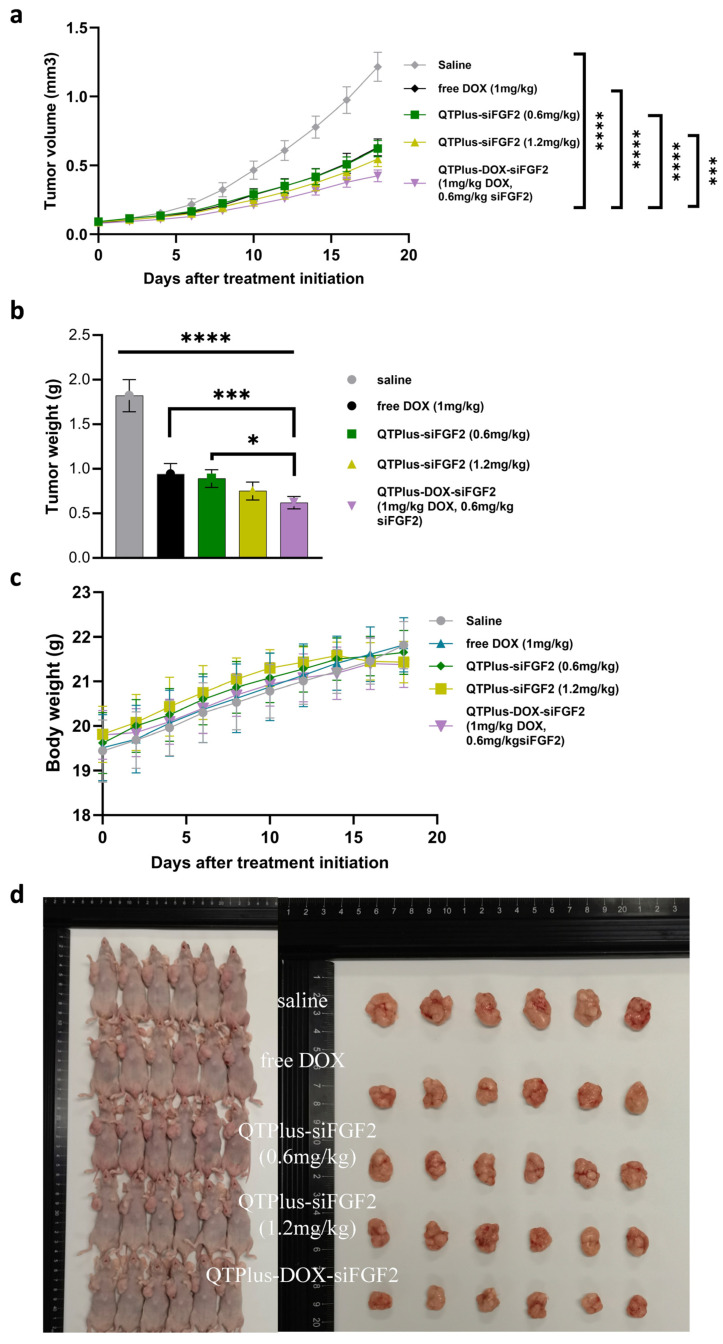
In vivo antitumor efficacy in the MDA-MB-231 xenograft model. (**a**) Changes in tumor volume throughout treatment course. (**b**) Tumor weight from mice at the end of study. (**c**) Body weight changes during the treatment period. (**d**) Representative photos of excised tumors at the end of the study. Mice were treated with saline (*n* = 12), free DOX (1 mg/kg, *n* = 12), QTPlus-siFGF2 (0.6 mg/kg, *n* = 6), QTPlus-siFGF2 (1.2 mg/kg, *n* = 6), or QTPlus-DOX-siFGF2 (1 mg/kg DOX, 0.6 mg/kg siFGF2, *n* = 6). Data are shown as mean ± SD. * *p* < 0.05, *** *p* < 0.001, **** *p* < 0.0001 vs. saline by Tukey’s HSD test after one-way ANOVA.

To evaluate the in vivo antitumor efficacy of the formulations, a subcutaneous xenograft model was established using MDA-MB-231 cells in female Balb/c-nu nude mice (4–6 weeks old). As shown in Figure 7a,b,d, the saline-treated group exhibited rapid tumor growth, with an average tumor volume of 1434 ± 129 mm^3^ by day 18. The free DOX and QTPlus-siFGF2 groups showed dose-dependent tumor growth suppression, with average tumor volumes of 756 ± 64 mm^3^ for the free DOX (**** *p* < 0.0001 vs. saline), 720 ± 69 mm^3^ for the 0.6 mg/kg dose (**** *p* < 0.0001 vs. saline) and 642 ± 74 mm^3^ for the 1.2 mg/kg dose (**** *p* < 0.0001 vs. saline). The QTPlus-DOX-siFGF2 group exhibited the most pronounced antitumor effect, with an average tumor volume of 502 ± 41 mm^3^ on day 18 (*** *p* < 0.001 vs. 1.2 mg/kg dose, **** *p* < 0.0001 vs. other groups). Tumor weights measured at the end of the study further confirmed these findings, with the saline group at 1.82 ± 0.18 g, free DOX at 0.94 ± 0.11 g, QTPlus-siFGF2 (0.6 mg/kg) at 0.89 ± 0.10 g, QTPlus-siFGF2 (1.2 mg/kg) at 0.75 ± 0.10 g, and QTPlus-DOX-siFGF2 at 0.62 ± 0.07 g (**** *p* < 0.0001 vs. saline, *** *p* < 0.001 vs. free DOX and * *p* < 0.0.05 vs. 0.6 mg/kg dose). The rationale for the different group sizes is provided in the Methods section. Statistical comparisons were performed using matched group sizes (*n* = 6), which confirmed the significant antitumor efficacy of the QTPlus-DOX-siFGF2 formulation.

To assess the safety and tolerability of the treatments, body weights were monitored daily throughout the 18-day study. In Figure 7c, the body weights of mice in the saline, free DOX and QTPlus-siFGF2 (0.6 mg/kg and 1.2 mg/kg) groups remained stable or showed slight increases, with mean weights of 22.0 ± 0.5 g, 22.0 ± 0.5 g, 21.9 ± 0.5 g, and 21.6 ± 0.5 g, respectively. The QTPlus-DOX-siFGF2 group experienced a slight decrease in body weight, with an average of 21.5 ± 0.4 g (*p* = 0.6963 vs. saline), suggesting minimal systemic toxicity within the acceptable range. Representative photos of excised tumors are shown in Figure 7d.

### 3.8. Histopathological Evaluation by H&E Staining

To evaluate morphological changes in major organs from mice with different treatments, H&E staining was performed on tumor, heart, liver, spleen, lung, and kidney sections harvested from each experimental group. In tumor sections (Figure S3), the saline group exhibited a densely packed tumor cell structure without visible interstitial gaps. In contrast, the free DOX group and QTPlus-siFGF2 (0.6 mg/kg) group showed scattered white intercellular clefts, suggesting partial necrotic regions or tumor loosening. More prominent white gaps were observed in the QTPlus-siFGF2 (1.2 mg/kg) group, indicative of enhanced tumor tissue disruption. Notably, the QTPlus-DOX-siFGF2 (1 mg/kg DOX, 0.6 mg/kg siFGF2) group displayed the most pronounced white areas, signifying extensive tumor architecture destruction and potential necrosis, consistent with enhanced antitumor activity.

In normal organs (Figure S3), cardiac tissue showed no notable morphological changes in any group, including the free DOX group at 1 mg/kg, indicating that the tested dose did not induce detectable structural cardiotoxicity. In hepatic tissue, the free DOX group exhibited mild red areas suggestive of localized inflammation, while other groups displayed no significant alterations. Splenic, pulmonary, and renal tissues showed only mild alterations with no significant differences compared to the saline group. These findings highlight the tissue-specific effects and favorable safety profile of the QTPlus-DOX-siFGF2 formulation.

### 3.9. Blood Biochemical Analysis

Safety was further assessed by serum biochemical analysis. Numerical trends toward lower cardiac injury markers (CK, CK-MB, LDH) were observed in the QTPlus-DOX-siFGF2 group compared with free DOX and high-dose QTPlus-siFGF2 monotherapy, although differences did not reach statistical significance in all pairwise comparisons after Tukey post hoc correction (detailed data in Supporting Information Figure S4). No significant changes were detected in hepatic (ALT, AST) or renal markers compared to the saline group.

## 4. Discussion

The development of a liposome-based co-delivery system for DOX and siFGF2 offers a promising approach to enhance the therapeutic efficacy of breast cancer treatment while addressing the limitations of conventional therapies, such as drug resistance and nonspecific toxicity [29,30,31,32]. The present study demonstrated that QTPlus-DOX-siFGF2 exhibited superior antitumor effects in both in vitro and in vivo models [5,33,34,35], with improved drug delivery efficiency and reduced systemic toxicity compared to free DOX, QTPlus-DOX and QTPlus-siFGF2. These findings align with recent advancements in combination nanotherapeutics, which have shown significant potential in overcoming the challenges of cancer treatment [33].

### 4.1. Physicochemical Properties and Encapsulation Efficiency

The physicochemical characterization of the liposomal formulations revealed that the QTPlus-DOX-siFGF2 formulation achieved a balanced particle size (218.18 ± 5.74 nm) and a low polydispersity index (PDI = 0.164), indicating a highly monodisperse and stable system [36]. The incorporation of siFGF2 likely contributes to the slightly larger size of QTPlus-DOX-siFGF2 compared to empty liposomes (eQTPlus) and QTPlus-DOX, as nucleic acids introduce additional steric and electrostatic interactions within the liposomal structure. The zeta potential of QTPlus-DOX-siFGF2 (+20.31 ± 3.18 mV) indicates a moderate positive charge, which enhances circulation stability by reducing liver uptake and nonspecific serum protein interactions, consistent with findings that optimizing positive charge density to approximately +15 mV significantly extends liposome blood circulation time compared to neutral liposomes. Additionally, cationic liposomes combined with PEG polymers improve in vivo stability and prolong blood circulation, further supporting enhanced delivery efficiency [37,38].

The EE% results highlight the superior performance of the A-066 ionizable lipid, achieving a DOX EE% of 49.56% and a siFGF2 EE% of 77.66%. These values are competitive with recent reports on liposomal systems, where encapsulation efficiencies for chemotherapeutic drugs and siRNA typically range from 40–60% [39] and 70–90% [40,41], respectively. While the simple co-injection method achieved a DOX EE% of 49.56%, which is sufficient for preclinical synergy with siFGF2, future optimizations could incorporate remote loading techniques (e.g., ammonium sulfate gradients) to exceed 90% EE%. This would further enhance clinical translatability without compromising siRNA co-encapsulation. The superior EE% of A-066 compared to tertiary amine lipids like SM-102 and MC3 stems from its cyclic amine (pyrrolidine) head group, a secondary amine structure that enhances hydrogen bonding, endosomal escape, and RNA delivery efficiency, outperforming tertiary amine lipids in vivo [42,43]. These enable efficient complexation with both DOX and anionic siFGF2, forming stable lipoplexes.

### 4.2. Mechanistic Insights into Drug Release Kinetics

The in vitro release data provide compelling mechanistic insights into the in vivo efficacy and safety of the QTPlus platform. The restricted DOX release at pH 7.4 (~30%) effectively prevents premature drug leakage in the bloodstream, which is consistent with the minimized systemic toxicity and cardiac safety observed in vivo. Upon encountering the acidic environment (pH 5.5), the ionizable lipid A-066 undergoes rapid protonation. This protonation increases the internal electrostatic repulsion between the positively charged DOX molecules and the cationic lipids, leading to structural dissociation and the rapid discharge of DOX (>81%).

While DOX exhibited profound pH-responsive release, the in vitro release of siFGF2 in acidic buffer was more conservative (reaching 36.92%). This is a recognized characteristic of ionizable lipid-nucleic acid complexes. In a cell-free PBS buffer at pH 5.5, the pronounced protonation of A-066 generates high positive charge density, which paradoxically strengthens the electrostatic binding with the polyanionic siRNA [44]. However, in vivo, endosomal escape and siRNA release do not rely solely on pH drop. It is widely established that within the endosomes, the protonated cationic lipids electrostatically interact and fuse with endogenous anionic lipids present in the endosomal membrane. This formation of ion pairs outcompetes the lipid-siRNA interactions, thereby displacing the siRNA and facilitating its efficient cytosolic release [45]. Therefore, despite the modest siFGF2 release observed in purely aqueous buffer, the robust down-regulation of FGF2 observed in our cellular assays confirms that the QTPlus platform effectively achieves endosomal escape and intracellular payload delivery.

### 4.3. Cellular Uptake and In Vitro Cytotoxicity

The comparison of five ionizable lipids (DODMA, SM-102, A-066, MC3, and ALC-0315) within the QTPlus-DOX-siFGF2 formulation highlights their differential impact on cellular uptake efficiency. The higher DiO fluorescence observed with ALC-0315 and MC3 suggests these lipids may enhance nanoparticle-membrane interactions, potentially due to their structural properties that promote endosomal escape [46,47,48]. Conversely, the elevated Cy5 signals for siFGF2 with DODMA and SM-102 indicate a stronger affinity for siRNA delivery, possibly linked to their cationic nature under physiological conditions [49,50,51]. DOX fluorescence was notably higher with DODMA and MC3, reflecting their potential to facilitate chemotherapeutic drug release, which may be attributed to differences in lipid pKa values affecting drug retention and release kinetics [52]. However, A-066 stood out for its balanced uptake profile across DiO, Cy5, and DOX, suggesting a versatile interaction with cellular components that minimizes trade-offs between siRNA and DOX delivery. This balanced performance positions A-066 as a promising candidate for optimizing QTPlus-DOX-siFGF2, aligning with the need for formulations that support both gene silencing and chemotherapy efficacy in a stable and reproducible manner [13].

The cellular uptake studies demonstrated that the QTPlus-DOX-siFGF2 formulation significantly enhanced DOX delivery (2.07-fold higher than QTPlus-DOX) while maintaining siFGF2 delivery efficiency comparable to the QTPlus-siFGF2 formulation. This synergistic effect likely stems from the co-delivery of both cargos to the same cellular compartment, enhancing their combined therapeutic impact. The high internalization of siRNA-loaded formulations (QTPlus-siFGF2 and QTPlus-DOX-siFGF2), as tracked by DiO fluorescence, suggests that siRNA encapsulation enhances the interaction between QTPlus and breast cancer cell membranes, possibly through electrostatic interactions [53]. The superior signal consistency of QTPlus-DOX-siFGF2 (Coefficient of Variation (CV) = 3.1%) compared to QTPlus-siFGF2 (CV = 15.5%) indicates that co-encapsulation stabilizes siRNA delivery, a critical factor for reproducible gene silencing in clinical applications.

The in vitro cytotoxicity of different formulations was evaluated using CCK-8 assays on MCF-7 and MDA-MB-231 cell lines following treatment with free DOX, QTPlus-DOX, and QTPlus-DOX-siFGF2. In MCF-7 cells, the IC50 values were 4.271 nM for free DOX, 25.37 nM for QTPlus-DOX, and 1.947 nM for QTPlus-DOX-siFGF2, indicating that QTPlus-DOX-siFGF2 exhibited the highest cytotoxicity, followed by free DOX, with QTPlus-DOX showing the lowest cytotoxicity. In MDA-MB-231 cells, the IC50 values were 927 nM for free DOX, 8846 nM for QTPlus-DOX, and 3253 nM for QTPlus-DOX-siFGF2, demonstrating that free DOX had the highest cytotoxicity, followed by QTPlus-DOX-siFGF2, with QTPlus-DOX exhibiting the lowest cytotoxicity. The observed lower cytotoxicity of QTPlus-DOX compared to free DOX in MDA-MB-231 cells may be due to controlled release from liposomes, which aligns with literature on encapsulation strategies [54].

In addition, flow cytometry analysis of apoptosis provided additional insights into the therapeutic efficacy of the formulations. The observed synergy can be mechanistically explained by complementary actions of the two agents. DOX primarily induces DNA damage and apoptosis, while siFGF2-mediated FGF2 silencing disrupts survival signaling (PI3K/AKT pathway) and EMT (VIM downregulation), thereby sensitizing cells to DOX. The higher potency of free DOX in short-term MDA-MB-231 cytotoxicity assays likely reflects immediate drug availability, whereas the liposomal formulation provides sustained release and simultaneous gene silencing, which translated to superior long-term in vivo efficacy (TGI 65.87% vs. 48.26% for free DOX).

RT-qPCR analysis further supported this synergy by demonstrating significant FGF2 downregulation (0.404 ± 0.077-fold for QTPlus-siFGF2 and 0.639 ± 0.175-fold for QTPlus-DOX-siFGF2), VIM downregulation, CASP3 upregulation, and BRCA1 upregulation, indicating that FGF2 silencing contributes to apoptosis induction and EMT suppression while complementing DOX-induced DNA damage responses.

The QTPlus-DOX-siFGF2 group exhibited the highest total apoptosis rate of 78.5%, significantly higher than that of the QTPlus-DOX group (42.65%) and the QTPlus-siFGF2 group (18.81%). The significant apoptosis induction in the QTPlus-DOX-siFGF2 group highlighted the synergistic effect of co-delivering DOX and siFGF2. It is well known that DOX induces apoptosis through DNA damage [25], and the silencing of FGF2 may further enhance this response by interfering with survival signals. This is consistent with previous studies showing that inhibition of FGF2 signaling can make cancer cells more sensitive to chemotherapeutic agents [55].

We acknowledge the concentration difference between the 24 h apoptosis assay (13 μM DOX) and the 72 h cytotoxicity assay (IC50 in the nM–μM range). This choice was made to ensure detectable apoptotic signals in a short timeframe while reflecting literature-reported peak tumor DOX levels. However, as noted in the Supplementary Data (Figure S2), the combination treatment at a lower concentration of 5 μM induced approximately 24.1% apoptosis. While this confirms the onset of programmed cell death at more moderate levels, the relatively modest response compared to the 13 μM group highlights a limitation of the 24-h assay timeframe. At lower concentrations, the synergistic interplay between FGF2-mediated chemosensitization and DOX-induced DNA damage likely requires a more extended period to fully execute the apoptotic cascade. This temporal dependency is consistent with our 72-h viability results, where significantly higher potency was achieved. Future dose–response apoptosis studies over extended timeframes and at physiologically lower concentrations (10–100 nM) are warranted to further characterize this synergy.

We also noted several results that warrant further mechanistic discussion. VIM downregulation was slightly stronger with QTPlus-DOX alone (0.453-fold) than with the combination (0.373-fold), possibly reflecting a dominant early effect of DOX on EMT at the 24 h time point, while siFGF2 contributes more to sustained inhibition via FGF2/PI3K/AKT pathways. QTPlus-siFGF2 alone induced only modest apoptosis (18.81%) despite robust FGF2 downregulation (0.404-fold), suggesting that FGF2 silencing primarily sensitizes cells to additional apoptotic triggers such as DOX rather than directly driving apoptosis. In MDA-MB-231 cells, free DOX showed a lower IC50 (927 nM) than QTPlus-DOX-siFGF2 (3253 nM); however, the nanoparticle formulation enables sustained release and simultaneous gene silencing, which translated to superior long-term in vivo tumor growth inhibition (65.87% TGI vs. 48.26% for free DOX).

### 4.4. In Vivo Antitumor Efficacy

The in vivo studies demonstrated that the QTPlus-DOX-siFGF2 formulation achieved the most pronounced antitumor effect in the MDA-MB-231 xenograft model [56], with a final tumor volume of 502 ± 41 mm^3^ compared to 1434 ± 129 mm^3^ in the saline group. This superior efficacy results from the synergistic action of DOX-induced cytotoxicity and siFGF2-mediated suppression of tumor growth and metastasis via FGF2 signaling inhibition. The free DOX group exhibited moderate tumor suppression, highlighting the enhanced therapeutic potency of the co-delivery system. The dose-dependent tumor suppression in the QTPlus-siFGF2 groups (0.6 mg/kg and 1.2 mg/kg) confirms the therapeutic potential of FGF2 silencing, consistent with its role in tumor invasiveness. Histological analysis of tumor sections further confirmed these observations. Tumors from the QTPlus-DOX-siFGF2 group displayed extensive structural disruption and interstitial gaps, indicating widespread tissue necrosis. The degree of disruption followed a dose-dependent trend, with the QTPlus-siFGF2 (1.2 mg/kg) group exhibiting more severe tissue disintegration than the 0.6 mg/kg group.

No significant weight loss was observed in all groups, indicating the safety of QTPlus-siFGF2-DOX. Histopathological examination revealed no detectable structural cardiotoxicity in cardiac tissue across all treatment groups. Biochemical analysis showed favorable numerical trends in cardiac biomarkers for the QTPlus-DOX-siFGF2 formulation. We therefore describe these findings as biochemical trends suggestive of improved cardiac safety rather than definitive reduction in cardiotoxicity, as structural evidence was absent in histology. These trends align with the known cardioprotective potential of liposomal encapsulation [57].

### 4.5. Clinical Implications and Future Directions

The co-delivery system presented in this study offers significant clinical potential for breast cancer treatment by combining DOX treatment with targeted FGF2 downregulation [58,59]. The enhanced efficacy and reduced toxicity observed in vitro and in vivo highlight the advantages of liposomal co-delivery over single-drug therapies. However, challenges such as enhancing tumor-specific targeting and ensuring long-term stability remain critical for clinical translation [60]. Future studies should explore surface functionalization with targeting ligands to improve tumor specificity and investigate the system’s applicability in other tumor models which highly express FGF2, such as lung or pancreatic cancer [61]. Additionally, comprehensive pharmacokinetic and biodistribution studies are needed to support scalability and clinical development.

In conclusion, the QTPlus-DOX-siFGF2 represents a promising strategy to overcome the limitations of conventional breast cancer therapies. By leveraging synergistic chemotherapy and gene silencing, this system achieves enhanced antitumor efficacy with improved safety, paving the way for advanced nanotherapeutics [62]. Future studies should explore surface functionalization with targeting ligands (e.g., folate or HER2 antibodies) to improve tumor specificity, scale-up under GMP conditions, and evaluate the system in patient-derived xenograft or orthotopic breast cancer models. Compared with recent LNP-based RNA therapies for breast cancer, QTPlus offers the advantage of balanced co-delivery of siRNA and chemotherapy with a favorable preclinical safety profile, supporting its potential for clinical translation [17,18].

### 4.6. Study Limitations

Several limitations of the present study should be acknowledged. Due to resource and ethical constraints, we did not include empty QTPlus (carrier-only), scrambled siFGF2, or non-encapsulated free DOX + free siFGF2 combination controls in the in vivo experiments. Consequently, we cannot completely exclude potential carrier-related effects or confirm that the observed synergy strictly requires nanoparticle-mediated co-delivery rather than simple mixing of the two agents. These essential controls will be incorporated in future studies. Additionally, gene silencing was assessed only at the mRNA level by RT-qPCR; protein-level validation (Western blot or immunofluorescence) will be performed to confirm functional changes in FGF2, CASP3, BRCA1, and VIM. The study relied on a single subcutaneous MDA-MB-231 xenograft model and did not include long-term toxicity assessments or orthotopic models, which limits generalizability. Future work will address these gaps through multi-model validation and extended safety studies.

Despite these limitations, the current data provide strong preclinical evidence for the therapeutic potential of QTPlus-DOX-siFGF2.

## 5. Conclusions

Although DOX is a cornerstone in breast cancer chemotherapy, its clinical application is hindered by severe cardiotoxicity and the emergence of multidrug resistance [63,64]. To address these limitations, combination therapies that incorporate DOX with siRNA targeting tumor-promoting genes like FGF2, which facilitates tumor growth, have been developed [61]. The present study introduced a QTPlus-based co-delivery system encapsulating doxorubicin (DOX) and siFGF2 for the treatment of breast cancer. The QTPlus-DOX-siFGF2 based on A-066 exhibited favorable physicochemical characteristics, including a uniform particle size, low polydispersity index, and high encapsulation efficiencies to both DOX and siFGF2. The system demonstrated highly pH-responsive drug release kinetics, tightly retaining therapeutic payloads at physiological pH to minimize systemic toxicity, while triggering rapid dissociation and release in acidic environments for optimal intracellular delivery. In vitro evaluations in MCF-7 and MDA-MB-231 cell lines demonstrated significantly enhanced cellular uptake of DOX and siFGF2 delivered by QTPlus. These results led to robust FGF2 gene silencing and modulation of key molecular pathways in vitro, including apoptosis activation and suppression of epithelial–mesenchymal transition [62]. In MDA-MB-231 xenograft model, the QTPlus-DOX-siFGF2 achieved superior antitumor efficacy with TGI% of 65.87%. Notably, this therapeutic advantage was achieved with minimal systemic toxicity, as evidenced by reduced cardiotoxic biomarkers and favorable histopathological findings in major organs compared with free DOX group (TGI% of 48.26%) [65]. These findings highlight the synergistic benefits of combining chemotherapeutic and gene-silencing modalities within a single nanocarrier, addressing key limitations of monotherapies such as drug resistance and limited therapeutic potency, and greatly benefiting patients with breast cancer.

## Figures and Tables

**Table 1:** Detailed compositions of QTPlus.

Components	Molar Ratio [%]
DOTAP	1
A-066	40
DSPC	5.6
Cholesterol	25.6
DMG-PEG 2000	1.2

## Data Availability

The data supporting the findings of this study are available from the corresponding author upon reasonable request. Certain datasets cannot be shared publicly due to ethical and privacy concerns related to sensitive information. For inquiries regarding data access, please contact zhongkunzhang@seu.edu.cn.

## Supplementary Materials

The following supporting information can be downloaded at: https://www.mdpi.com/article/10.3390/pharmaceutics18050589/s1, Figure S1. Comprehensive comparison of drug release kinetics across varying pH conditions. (a) Comparison of cumulative DOX release among all tested groups at pH 7.4 and pH 5.5. (b) Direct comparison of DOX release kinetics between Free DOX and the dual-loaded QTPlus-DOX-siFGF2 system under both pH conditions. (c) Comparison of cumulative siFGF2 release among all tested groups at pH 7.4 and pH 5.5. (d) Direct comparison of siFGF2 release kinetics between Free siFGF2 and the dual-loaded QTPlus-DOX-siFGF2 system under both pH conditions. Data are presented as mean ± SD (*n* = 3). Figure S2. Flow cytometry analysis of cell apoptosis in different treatment groups. The table presents the percentages of cells in each quadrant: UL (necrotic cells), UR (late apoptotic cells), LL (viable cells), LR (early apoptotic cells), and the total apoptosis percentage for the Untreated, QTPlus-DOX, QTPlus-siFGF2, and QTPlus-DOX-siFGF2 groups. Figure S3. Representative H&E-stained tissue sections from treated animals. Histological evaluation of tumors and major organs (heart, liver, spleen, lung, kidney) from mice treated with saline, free DOX (1mg/kg), QTPlus-siFGF2 (0.6 mg/kg), QTPlus-siFGF2 (1.2 mg/kg), and QTPlus-DOX-siFGF2 (1mg/kg DOX, 0.6 mg/kg siFGF2). Scale bar = 2 mm and 200 μm. Figure S4. Blood biochemical analysis to assess systemic toxicity in mice treated with saline, free DOX (1mg/kg), QTPlus-siFGF2 (0.6 mg/kg), QTPlus-siFGF2 (1.2 mg/kg), and the QTPlus-DOX-siFGF2 (1mg/kg DOX, 0.6 mg/kg siFGF2). Levels of (A) ALT, (B) AST, (C) TG, (D) T-CHO, (E) CK, (F) CK-MB, (G) LDH, and (H) LDH1 were measured by Automatic Biochemical Analyzer (Rayto Life Science Co., Ltd., China). Data are presented as mean ± SD. (*n*=6 per group). Statistical analyses were performed using one-way ANOVA followed by Tukey’s post-hoc test. * *p* < 0.05, ** *p* < 0.01, *** *p* < 0.001 vs. saline or indicated group. Table S1. The forward and reversed primer sequences. Table S2. Representative compositions and molar ratios of clinically approved or representative lipid nanoparticle (LNP) platforms.
