# Supplementary material for: Synergistic Co-Delivery of siFGF2 and Doxorubicin via QTPlus Nanoparticles for Enhanced Breast Cancer Therapy
Source: Pharmaceutics. 2026 May 10;18(5):589. doi: 10.3390/pharmaceutics18050589 (PMC13210936; doi:10.3390/pharmaceutics18050589)
Supplement: Supplementary file 1 [file pharmaceutics-18-00589-s001.zip › pharmaceutics-4254686-supplementary.pdf]

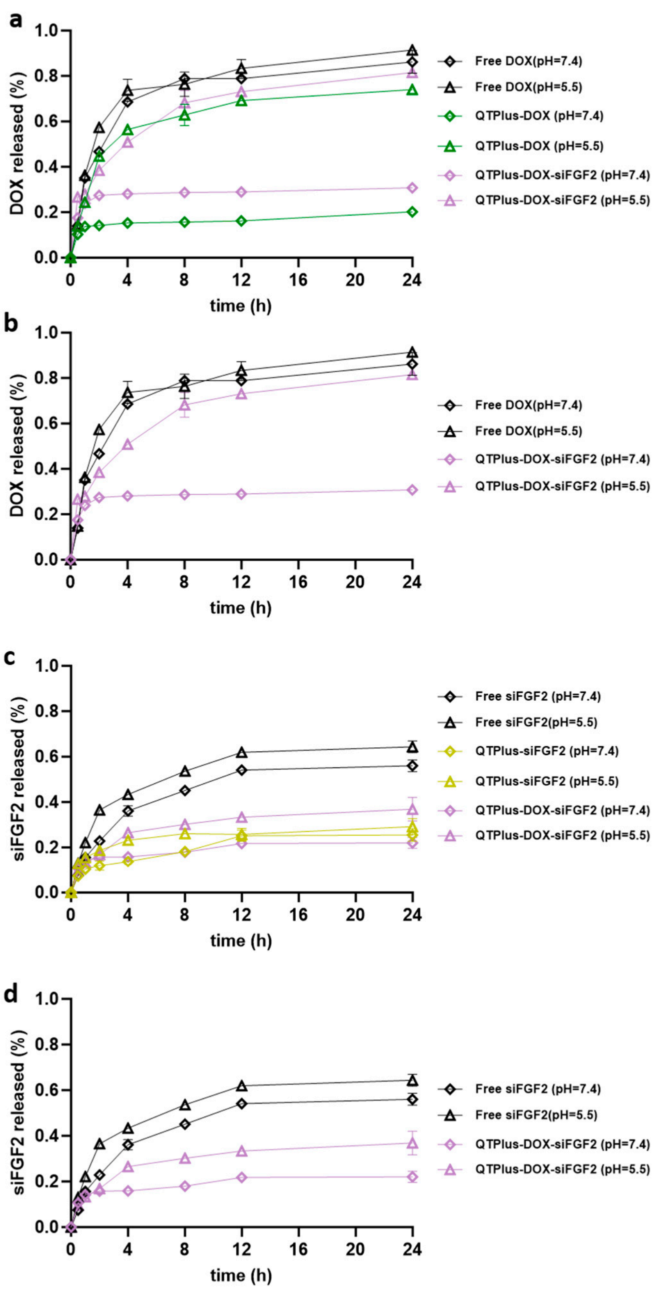

Figure S1. Comprehensive comparison of drug release kinetics across varying pH conditions. (a) Comparison of cumulative DOX release among all tested groups at pH 7.4 and pH 5.5. (b) Direct comparison of DOX release kinetics between Free DOX and the dual-loaded QTPlus-DOX-siFGF2 system under both pH conditions. (c) Comparison of cumulative siFGF2 release among all tested groups at pH 7.4 and pH 5.5. (d) Direct comparison of siFGF2 release kinetics between Free siFGF2 and the dual-loaded QTPlus-DOX-siFGF2 system under both pH conditions. Data are presented as mean ± SD (n = 3).

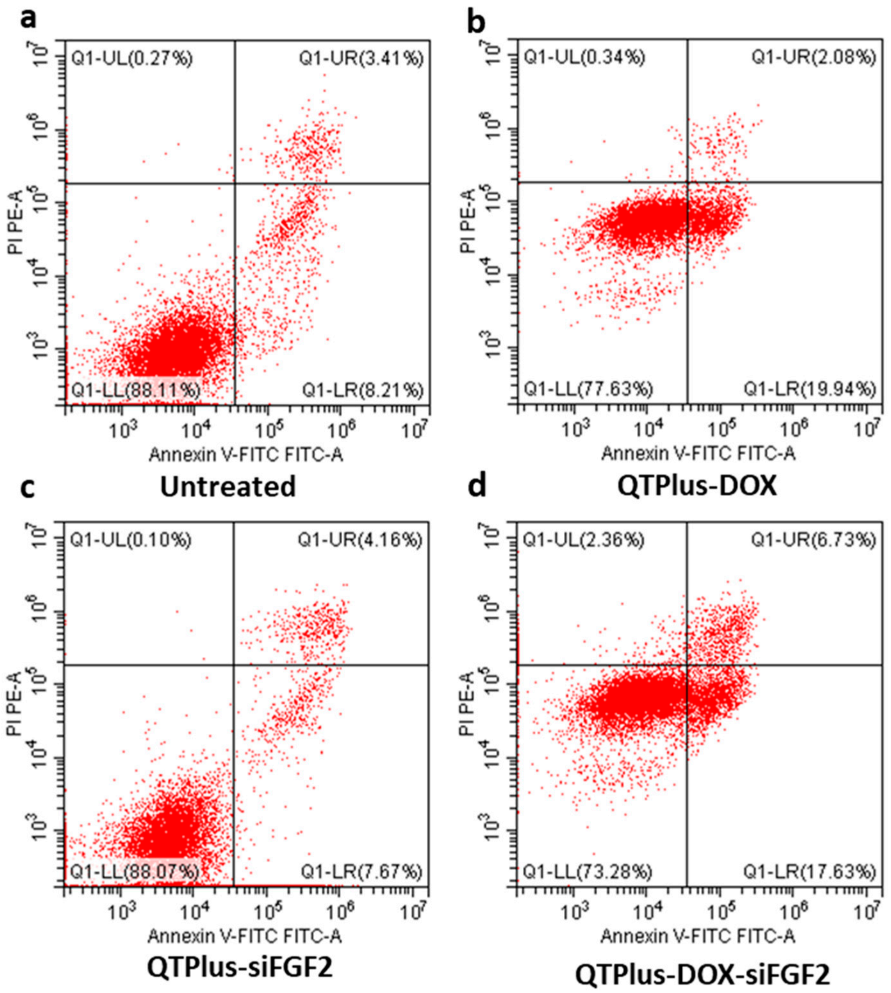

Figure S2. Flow cytometry analysis of cell apoptosis in different treatment groups. The table presents the percentages of cells in each quadrant: UL (necrotic cells), UR (late apoptotic cells), LL (viable cells), LR (early apoptotic cells), and the total apoptosis percentage for the Untreated, QTPlus-DOX, QTPlus-siFGF2, and QTPlus-DOX-siFGF2 groups.

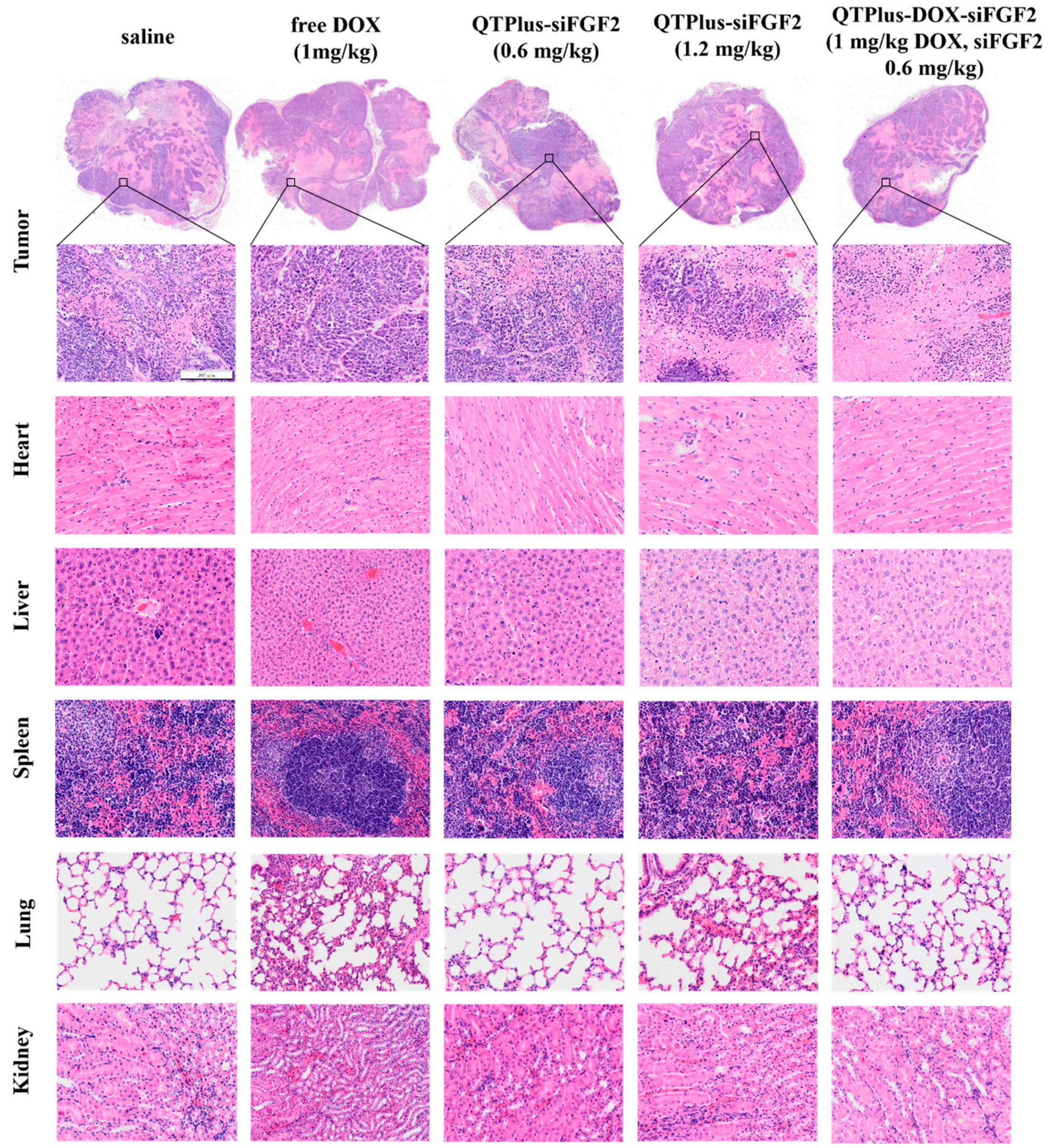

Figure S3. Representative H&E-stained tissue sections from treated animals. Histological evaluation of tumors and major organs (heart, liver, spleen, lung, kidney) from mice treated with saline, free DOX (1mg/kg), QTPlus-siFGF2 (0.6 mg/kg), QTPlus-siFGF2 (1.2 mg/kg), and QTPlus-DOX-siFGF2 (1mg/kg DOX, 0.6 mg/kg siFGF2). Scale bar = 2 mm and 200 μm.

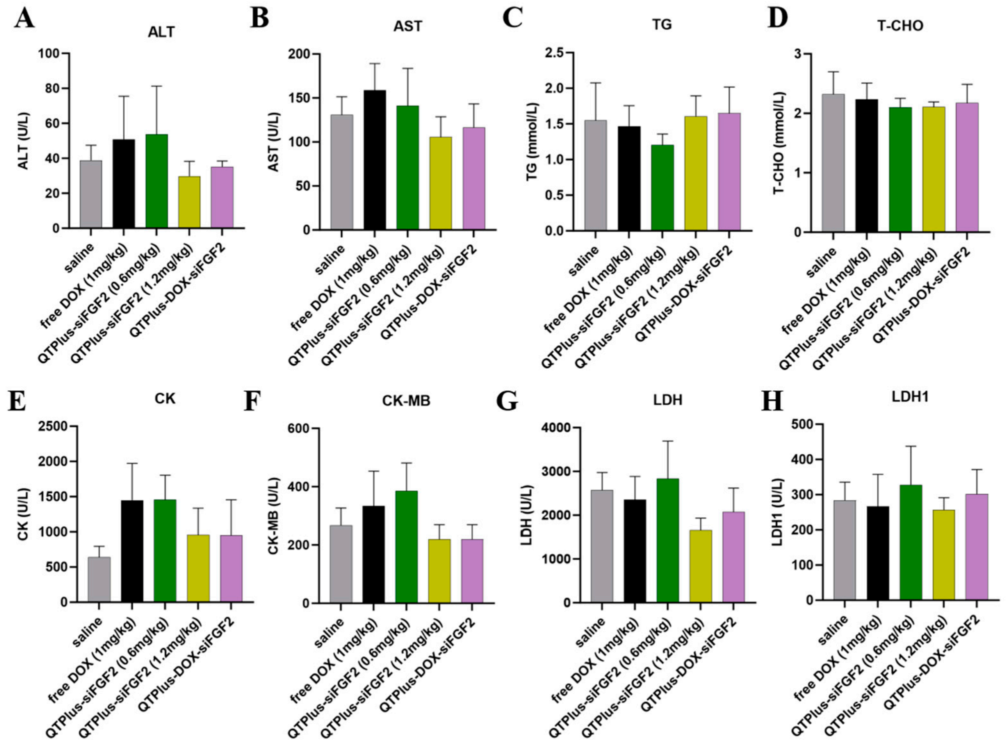

Figure S4. Blood biochemical analysis to assess systemic toxicity in mice treated with saline, free DOX (1mg/kg), QTPlus-siFGF2 (0.6 mg/kg), QTPlus-siFGF2 (1.2 mg/kg), and the QTPlus-DOX-siFGF2 (1mg/kg DOX, 0.6 mg/kg siFGF2). Levels of (A) ALT, (B) AST, (C) TG, (D) T-CHO, (E) CK, (F) CK-MB, (G) LDH, and (H) LDH1 were measured by Automatic Biochemical Analyzer (Rayto Life Science Co., Ltd., China). Data are presented as mean ± SD. (n=6 per group). Statistical analyses were performed using one-way ANOVA followed by Tukey's post-hoc test. *$p < 0.05$, **$p < 0.01$, ***$p < 0.001$ vs. saline or indicated group.

Table S1. The forward and reversed primer sequences

Gene	Forward sequence	Reverse sequence
Gapdh	5'-GACCCCTTCATTGACCTCAAC -3'	5'-CTTCTGAGTGGCAGTGATGG -3'
FGF2	5'-CAGCCACATCAAGACTACAGC -3'	5'-GGCTCTTAGCAGACATTGGAAGA -3'
Caspase-3	5'-AGAACTGGACTGTGGCATTG -3'	5'-ATCTGTTGCCACCTTTCGGT -3'
BRCA1	5'-GCTGCTCAGGAAAGGTTTTT -3'	5'-AAGTTGGTTGTTGGGTTTGG -3'
Vimentin	5'-AGTCCACTGAGTACCGGAGAC -3'	5'-CATTTCACGCATTGCCTTCC -3'

Table S2. Representative compositions and molar ratios of clinically approved or representative lipid nanoparticle (LNP) platforms

Platform / Product	Ionizable Lipid	Helper Lipid	Sterol	PEG-Lipid	Molar Ratio (mol %)
Alnylam (Onpattro)	DLin-MC3-DMA	DSPC'	Cholesterol	DMG-PEG 2000	50/10/38.5/1.5
Pfizer/BioNTech (BNT162b2)	ALC-0315	DSPC	Cholesterol	ALC-0159	46.3/9.4/42.7/1.6
Moderna (mRNA-1273)	SM-102	DSPC	Cholesterol	DMG-PEG 2000	50/10/38.5/1.5
